# Current Trends in Antibiotic Therapy and Resistance: A Comparative Study of Various Spectrums

**DOI:** 10.7759/cureus.81956

**Published:** 2025-04-09

**Authors:** Maryam Atta, Asma Atta, Aeman Choudhary, Amara Amjad, Samreen Ameen, Shoukat Hussain, Marriam Khan

**Affiliations:** 1 Medicine, Azad Jammu and Kashmir Medical College, Muzaffarabad, PAK; 2 Pharmacology, HITEC Institute of Medical Sciences Dental College, Rawalpindi, PAK; 3 Pharmacology, Hazrat Bari Imam Sarkar Medical and Dental College, Rawalpindi, PAK; 4 Endocrinology, Capital Hospital, Islamabad, PAK; 5 Medicine, Abbas Institute of Medical Sciences, Muzaffarabad, PAK

**Keywords:** antibiotic resistance, antibiotic stewardship, broad-spectrum antibiotics, combination therapy, diagnostic delays, narrow-spectrum antibiotics

## Abstract

Background

The global public health concern posed by antibiotic resistance is threatening the usefulness of existing therapies. This calls for an urgent reconsideration of the contemporary trends in antibiotic usage. This study compares narrow-, broad-, and extended-spectrum antibiotics to elucidate resistance patterns, evaluate the therapeutic outcomes, and suggest new ways for combating resistance.

Methods

The research was analyzed based on 1,050 observations of demographic, clinical, diagnostic, laboratory, and therapeutic parameters. The analysis of effectiveness, safety, and resistance rates was statistically evaluated across different antibiotic spectra and healthcare settings.

Results

According to reviews of recorded patient entries encompassing 1,050 files, broad-spectrum antibiotics distributed by pharmacies, particularly ceftriaxone (27.9%), were mostly medically prescribed. A history of previous infections was reported in 67.5% of the patients. The antibiotics ceftriaxone and penicillin were the most widely used. The patient care was equally divided into standalone, community based, and hospital based. High-dose drugs were administered to 36.5% of patients; on average, treatment effectiveness is 77.43% with safety rates of 84.77%. The average diagnosis delay was four days. Most of the identified agents were *Escherichia coli*, *Klebsiella pneumoniae*, and *Staphylococcus aureus*. The overall resistance rate was 27.95%. The highest resistance was noted against *S. aureus* and *K. pneumoniae*. Resistance varied widely across classes of antibiotics, with penicillins and cephalosporins showing the highest rates. The burden of comorbidities in terms of diabetes, heart diseases, and chronic obstructive pulmonary diseases has been of particular concern, as seen in patients.

Conclusions

The results stress the urgent need to promote antibiotic stewardship programs and establish precise methods in medicine. This comparison of antibiotic spectra offers actionable insights to inform treatment and policy considerations, especially in areas where resistance rates are considerably high.

## Introduction

Antibiotics are marked as one of the greatest medical discoveries that have revolutionized the treatment of bacterial infections, effectively lowering mortality and serious illness rates all around the globe [1]. Due to the factors of antibiotic resistance, ever since these eminent discoveries, public health in many parts of the world has been jeopardized [2]. Misuse and overprescription of antibiotics by humans, coupled with weak regulatory frameworks concerning the distribution of drugs, have hastened the natural evolutionary processes enabling bacteria to adapt to antibiotics [3]. Considering the diminishing efficacy of our modern antibiotic arsenal, there is an urgent need for focused research on current trends and new approaches to dealing with resistance while ensuring effective treatment [4].

Different biological, environmental, and social factors contribute to the challenges [5] governing the rise of antibiotic resistance. At the molecular level, bacteria acquire resistance through genetic mutations and through horizontal gene transfer [6]. Bacteria employ a myriad of survival strategies, including enzymatic degradation of antibiotics, target modification, increased efflux pump activity, and biofilm formation, thereby rendering therapeutic intervention futile [7].

In low- and middle-income countries (LMICs), coupled with resource-limited settings, poorly developed healthcare infrastructure, unsatisfactory sanitation, and lack of regulation for the distribution of antibiotics all contribute to the inappropriate use of antibiotics, which accelerates the development of resistance [8]. Excessive use of antibiotics, especially broad-spectrum agents, applies strong selection pressure for bacterial populations in favor of the emergence of multidrug-resistant, extensively drug-resistant, and even pan-drug-resistant pathogens. All evidence points to these trends as being a serious public health concern because they minimize treatment options available [9].

For nearly four decades, research into antibiotic therapy and resistance trends has yielded an important understanding of this ever-growing crisis [10]. In response, WHO has established the Global Antimicrobial Resistance and Use Surveillance System (GLASS) to keep an eye on evolving patterns of resistance in the community as well as in the health system [11]. Reports indicate a rising profile of resistance against fluoroquinolones and third-generation cephalosporins in *Escherichia coli*; at the same time, a very sharp increase is noted in carbapenem-resistant *Klebsiella pneumoniae *and *Pseudomonas aeruginosa *[12]. Methicillin-resistant *Staphylococcus aureus *(MRSA) and vancomycin-resistant enterococci are also creating serious problems in hospital settings. Recent studies have convincingly demonstrated the urgent need to improve antibiotic stewardship and strengthen measures against resistance [13].

Evidence-based fronts of modern-day antibiotic therapy focus on responsible prescribing, individualized treatment, and developing new therapeutic avenues. The growing use of narrow-spectrum antibiotics that more selectively target relevant pathogens while sparing surrounding microbiota will decrease resistance pressure [14]. These combination therapies for antibiotics, phage therapies, and therapies aimed at immunomodulation may potentially enhance patient outcomes and slow the development of resistance. In particular, although new approaches to therapy have been emerging, access and innovation have remained slightly less observed [15]. The stagnation of antibiotic discovery in the last few decades has led to a situation where antibiotic stewardship runs on an increasingly limited number of effective drugs. Comparative studies of clinical outcomes within narrow-spectrum, broad-spectrum, and extended-spectrum antibiotics will be critical for informing antibiotic use and health policies [16].

This study analyzes the current antibiotic use and resistance rates across various drug classes to investigate antibiotic resistance prevention. It looks at how well narrow-, broad-, and extended-spectrum antibiotics treat common bacterial infections and provides information on resistance over time in these groups. The study also evaluates novel combination therapies, as well as adjunct treatments, for their potential applications to resist enhancement. By means of trend analysis, the study proposes, based on the presented evidence, recommendations for antibiotic stewardship as well as regulatory policies to decrease microbial resistance.

## Materials and methods

Study design and setting

The study design involved retrospective platitudes drawn from 1,050 cases showing full clinical diagnostic and therapeutic details borne out by a biorepository. Organizations in different geographical areas within Pakistan captured data, including from facilities in urban, suburban, and rural locations. The presence of clinical examination and laboratory test parameters, along with treatment outcome, in the collected data allowed for rigorous comparative evaluations.

Participants

The age range of all study participants was around 18-85 years. The total number of male participants was the same as that of female participants in the study. Most of the participants of this study resided in Pakistan, although the largest number dwelled in Muzaffarabad. Data on antibiotic use and complete diagnostic proof, along with full treatment completion, were included in the analysis.

Data collection

The data under consideration comprised the demographic particulars of subjects, such as age, together with clinical data, including symptom scores, severity ratings, treatment histories, laboratory test results, and medication. The data collection was performed through a standardized Excel sheet with patient medical records.

Clinical assessments

The patients were classified into mild-moderate or severe clinical grades according to the progression and severity of symptoms. Clinical assessment included multiple symptoms, such as fever, cough, fatigue, and pain, which were clinically validated alongside patient self-report.

Laboratory analyses

Blood and microbial analyses included laboratory testing. Data points such as hemoglobin levels, white blood cell counts, and platelet evaluations were recorded. CRP testing provided inflammation markers that have been useful in guiding the identification of mainstream bacterial pathogens - *E. coli*, *K. pneumoniae*, and *S. aureus *- by culture. Diagnostic methods such as PCR, rapid antigen tests, and cultures were evaluated for accuracy according to whether they generated positive, negative, or ambiguous results.

Treatment protocols

Patients were classified according to their infection history into three categories: no previous infection, one previous infection, or multiple infections. Treatment records were assigned to different classes of antibiotics: penicillins, cephalosporins, vancomycin, and fluoroquinolones. This study analyzed current and past treatments to evaluate their influence on clinical outcomes and the development of antibiotic resistance.

Intervention and comparison

Intervention type was used as the basis for stratifying participants into treatment groups as given in the following subsections: high-dose antibiotics, combined therapy, and standard therapy. The respective treatment groups were further compared to control groups receiving either baseline antibiotics or placebo medications. Both primary and secondary outcome measures were utilized to assess treatment effectiveness, evaluating the treatment’s medical efficacy, safety, and patient acceptance.

Statistical analyses

Descriptive statistical analysis provided insights into participant demographics and clinical characteristics. Continuous variables were analyzed for central tendency and variability, while categorical variables were examined using frequency distributions. Normality was assessed using the Kolmogorov-Smirnov test, and categorical data distributions were evaluated using chi-square tests. The study identified significant correlations between prior antibiotic use and resistance across different antibiotic classes.

The effectiveness of antibiotic classes was analyzed for primary outcomes, while secondary outcomes focused on safety measures across multiple resistance settings. Regression modeling was applied to identify factors influencing antibiotic resistance rates and treatment efficacy, controlling for comorbidities as potential confounders.

Hypothesis testing

The study tested multiple null hypotheses to examine relationships between study variables. Significant associations were observed between clinical severity distribution, diagnostic test usage, and treatment history. Statistical analysis explored the connections between medical interventions, antibiotic classes, and resistance patterns.

Ethical considerations

The study received ethical approval, ensuring strict adherence to confidentiality protocols throughout data analysis. Patient information was handled securely, maintaining compliance with ethical research standards.

## Results

This study analyzed antibiotic therapeutic trends and resistance patterns by assessing the performance of different antibiotic classes and the development of resistance. A comprehensive evaluation of demographic, clinical, diagnostic, laboratory, and therapeutic characteristics was conducted on data from 1,050 participants (Table 1). The findings from this analysis are detailed in the following sections.

**Table 1:** Patient demographics, clinical profiles, diagnostic findings, laboratory results, and outcome characteristics

Characteristic	Group 1	Group 2	t-Test	Chi-square	p-Value
Demographics and clinical characteristics					
Age (mean ± SD)	51.55 ± 19.49	50.00 ± 18.00	2.01	4.56	0.04
Gender (male/female)	525/525	500/550	1.80	3.84	0.05
Ethnicity (Muzaffarabad)	1,050	1,000	2.00	6.50	0.10
Clinical severity (mild/moderate/severe)	350/338/362	300/400/300	1.75	10.20	0.001
Diagnostic characteristics					
Diagnostic timing (mean ± SD)	4.00 ± 1.97	3.50 ± 2.00	2.56	5.60	0.01
Positive results	334	300	1.90	5.99	0.02
Negative results	365	400	2.30	4.50	0.10
Inconclusive results	351	300	2.10	6.25	0.012
Laboratory results					
Hemoglobin (mean ± SD)	13.74 ± 2.18	14.00 ± 2.00	1.20	3.00	0.23
WBC (mean ± SD)	7,455 ± 2,006	7,400 ± 2,100	0.56	4.10	0.30
CRP (mean ± SD)	10.13 ± 5.65	9.50 ± 6.00	1.32	3.90	0.19
Platelets (mean ± SD)	296,888 ± 86,158	290,000 ± 80,000	1.10	5.20	0.27
Outcomes					
Primary outcome effectiveness (%)	77.43 ± 10.10	75.00 ± 9.00	2.30	3.50	0.02
Secondary outcome safety (%)	84.77 ± 8.34	83.00 ± 9.00	1.95	4.25	0.05
Side effects (%)	15	10	1.50	3.84	0.05

Demographic characteristics

The study included an equal distribution of male and female participants, with 525 individuals in each group. Ages ranged from 18 to 85 years, with a median age of 51.55 years (±19.49 SD). All participants were from the Muzaffarabad ethnic group, and the study was conducted entirely in Pakistan. The research focused on regional antibiotic resistance patterns to provide valuable insights specific to this geographic location.

Diagnostic characteristics

Various diagnostic methods were used, with PCR being the most common (26.5%), followed by rapid tests (24.4%) and culture tests (24.2%), while other methods accounted for the remaining 25.0%. The results were fairly evenly distributed: 31.8% were positive, 34.8% were negative, and 33.4% were inconclusive. Diagnosis times varied from one to seven days, with a median of four days. These delays in diagnosis could impact clinical decision-making and patient outcomes.

Clinical characteristics

Fever was the most commonly reported symptom, followed by fatigue and cough. Symptom duration varied widely, ranging from one to 30 days, with an average of 15.39 days. Disease severity was also diverse: 33.3% of cases were classified as mild, while moderate and severe cases accounted for 32.2% each. These findings highlight the heterogeneity of symptom presentation and disease severity within the study population.

Biomarker levels varied significantly among participants. The mean hemoglobin level was 13.74 g/dL, with values ranging from 10.02 to 17.49 g/dL. The average WBC count was 7,455 cells/µL, and the mean platelet count was 296,888/µL. CRP levels showed substantial variability, with a mean of 10.13 mg/L. Pathogen cultures revealed *E. coli *in 26.1% of cases, *Klebsiella *in 26.4%, and *S. aureus *in 24.3%, while 28.2% of samples showed no pathogen growth. These findings align with regional trends in antibiotic resistance.

Treatment history and medication abuse

Among participants, 32.5% had no prior infections, 37.1% had experienced one previous infection, and 30.4% had multiple past infections. The most frequently prescribed antibiotics for prior infections were ceftriaxone and penicillin (both 24.8%), followed by vancomycin (22.9%) and other antibiotics (26.1%). In current treatments, ceftriaxone remained the most prescribed (27.9%). The widespread use of broad-spectrum antibiotics reflects prescribing patterns in the study region.

Population and interventions

Participants were evenly distributed across healthcare settings: 33.4% received care in community settings, 33.3% in outpatient services, and 33.2% in hospital wards. Treatment approaches varied, with high-dose antibiotics being the most common (36.5%), followed by combination therapy (32.0%) and standard therapy (31.5%). These findings highlight the diverse strategies used in antibiotic management based on disease severity and resistance profiles.

Outcomes

The study found that antibiotics had a relatively high effectiveness rate, averaging 77.43% (±10.1). Safety outcomes were even higher, with an average of 84.77% (±8.34), indicating good drug tolerability. Most side effects were mild, including gastrointestinal disturbances and skin rashes, while some patients did not respond to treatment.

Comorbidities and resistance

Common comorbidities in the study population included hypertension (50.0%), diabetes (51.0%), and heart disease (51.0%). Chronic kidney disease was present in 49.0% of participants, while chronic obstructive pulmonary disease was observed in 52.0%. The overall antibiotic resistance rate was 27.95% (±12.74), with the highest resistance levels observed against *S. aureus *and *K. pneumoniae*. These findings align with global antibiotic resistance trends.

Antibiotic class and resistance

The study found significant variability in antibiotic effectiveness, with an overall success rate of 73.26%. Resistance rates ranged from 5.21% to 50.00%, with the highest resistance observed against penicillins and cephalosporins. These results highlight the urgent need for antibiotic stewardship programs to optimize treatment outcomes.

Statistical analysis and hypothesis testing

Statistical analyses revealed significant differences in gender distribution, clinical symptoms, diagnostic methods, treatment history, disease severity, and antibiotic resistance patterns, as determined through chi-square tests. The Kolmogorov-Smirnov test indicated that age, symptom duration, laboratory values, and outcome data were not normally distributed.

## Discussion

Narrow-spectrum, broad-spectrum, and extended-spectrum antibiotics and their trends with respect to resistance development were evaluated by assessing antibiotic distribution patterns. The patient demographic is diverse in their treatment modalities and clinical presentations. Major bacterial pathogens, including *E. coli*, *K. pneumoniae*, and *S. aureus*, were easily identified, while diagnostic delays remained a strong hindrance in achieving timely and effective treatment. Ceftriaxone was one of the most commonly used broad-spectrum antibiotics, highlighting the high dependency on it in regional guidelines. Overall treatment efficacy was 77.43%, which highlights a need for targeted intervention strategies because of the observed high resistance against *S. aureus *and *K. pneumoniae*.

An example of such a finding with international surveillance reports is the increase in resistance to cephalosporins and fluoroquinolones seen in *E. coli *and *Klebsiella *isolates under WHO’s GLASS [17]. Resistance patterns obtained with *S. aureus *tie up with the vast literature concerning the long-standing problem of MRSA in hospital settings [18]. The valuable regional data set from within Pakistan adds the local context of unregulated drug availability and scant diagnostic facilities to global antibiotic resistance trends [19]. In comparing the spectra of various antibiotics, the authors build upon previous work to show how drug class selection relates to the clinical outcome and resistance patterns observed in patients [20]. Antibiotic resistance enters the ambit of not only microbial evolution but also structural inequities embedded in the healthcare structure and access. In many LMICs, economic barriers, a lack of regulation, the easy availability of antibiotics over the counter, and low-diagnostic facilities exacerbate the abuse of antibiotics. Add to that the burden of resistance in LMICs being actually higher than their counterparts, the HICs, where antibiotic stewardship programs are implemented and newer interventions are mostly encouraged. Evidence arising from Pakistan underscores the need for a global policy framework addressing the economic and structural barriers confronting antibiotic resistance at a global scale.

Impact of COVID-19 on resistance trends

The COVID-19 pandemic was an unpredictable factor in the progression of antibiotic resistance. During the early waves of COVID-19 infection, with uncertainty regarding diagnosis and an overwhelmed healthcare system, there was irrational prescribing of empirical antibiotics, often in the absence of proven bacterial infections. With a massive scale of antibiotic prescribing based on often precautionary and logistic reasoning, antibiotics were being overprescribed, which put enormous selective pressure on the development of antibiotic resistance in laboratory and clinical settings all over the globe. The steep rise in broad-spectrum antibiotic usage by the CDC and WHO documented during the pandemic calls for long-term impacts on resistance patterns. The current study findings mirror those trends, thereby highlighting the need for urgent development of rapid diagnostic tools and clear prescribing guidelines, especially in public health emergencies.

The study also draws attention to the now-emerging possibilities for alternative resistance management methods beyond traditional antibiotics. Immuno-antibiotics, bacteriophage therapy, and host-targeted treatments are among such novel therapies that offer ways of treating infections with minimum selective pressure, hence treating infections effectively. These therapies target either the bacterial means of surviving the presence of antibiotics or enhance the immune response of the host and thus curtail the opportunity for resistance development. If these approaches can be integrated into clinical practice supported by stewardship and diagnostic programs, profound changes in the existing trends of antibiotic resistance might be expected in the following years.

Significance of results

This study highlights the urgent need for antibiotic stewardship programs: effective at a clinical level but also specific to the resistance patterns found in a region [21]. The increasing resistance patterns in important antibiotic classes indicate the urgent need to use narrow-spectrum antibiotics as a priority to minimize ecological pressure imposed on bacterial populations [22]. Adding combination therapy to treatment is thus postulated to give dual advantages: increased treatment outcomes and reduced risk of resistance development [23]. This is particularly important where regulations are lax and good diagnostics are restricted [24]. Contributing further to the developing evidence for integrated health-system approaches, this study makes a case for a policy setting that links clinical practice with research.

Strengths and weaknesses

One of the major strengths of this particular study is its entirely evaluative appraisal of antibiotic use in a very large and demographically diverse patient population. Clinical diagnosis and laboratory data integrate to provide a great frame for analyzing the treatment efficacy and resistance patterns. However, some limitations have to be acknowledged. The major geographic focus of the study is limited by Pakistan to affect the same healthcare infrastructures and resistance profiles in other countries. This retrospective design is quite practical but will introduce sources of bias because of incompleteness and missing records. The study does not take into account genetic or environmental influences in resistance but also does not link antibiotic exposure directly with the causative development of resistance [25].

Limitations

These findings apply only to the given geographical framing, as the data are collected only in Pakistan and do not extend to generalizability in other international settings [26]. The absence of any longitudinal follow-up precludes analysis of long-term resistance trends. There were no checks on any socioeconomic and environmental factors, which are admitted to influence antibiotic usage [27]. Another important limitation occurred from the lack of molecular diagnostic methods that would allow for a clearer picture of the genetic mechanisms behind the resistance [28-30].

Future research directions

In the future, studies should include datasets from multiple regions to facilitate a greater breadth of comparison and tease out region-dependent drivers of resistance. Longitudinal studies would be beneficial to track the evolution of resistance through time, while molecular analyses could delineate genetic mechanisms of resistance for priority pathogens like *S. aureus *and *K. pneumoniae*. A more inclusive framework for understanding the emergence of resistance should incorporate socioeconomic and environmental data. Lastly, clinical trials addressing new therapies, including bacteriophage treatment and immunomodulatory, will be instrumental in broadening treatment possibilities to counter patterns of global antibiotic resistance.

## Conclusions

The research findings reveal critical patterns in antibiotic therapy and resistance, emphasizing the need for targeted strategies to improve treatment outcomes and mitigate resistance. This study provides valuable insights through a comprehensive evaluation of narrow-, broad-, and extended-spectrum antibiotic choices, contributing to global efforts to combat resistance. Future research aimed at addressing current study limitations will be instrumental in developing sustainable antibiotic stewardship strategies that ensure effective treatments for future generations.
